# Is there benefit from the use of cochlear implants and hearing aids in cognition for older adults? A systematic review

**DOI:** 10.3389/fepid.2022.934750

**Published:** 2022-08-30

**Authors:** Natalia Carasek, Pauliana Lamounier, Isabela Gomes Maldi, Marina Nahas Dafico Bernardes, Hugo Valter Lisboa Ramos, Claudiney Cândido Costa, Fayez Bahmad

**Affiliations:** ^1^Center for Rehabilitation and Readaptation Dr. Henrique Santillo (CRER), Goiânia, GO, Brazil; ^2^Post Graduate Program of the Faculty of Health Sciences of the University of Brasília, Brasília, DF, Brazil

**Keywords:** cochlear implants, hearing aids, correction of hearing impairment, cognition, cognition disorders

## Abstract

**Objectives:**

The aim of the study was to assess whether hearing aids (HA) and cochlear implants (CI) bring benefits to cognition or mitigate cognitive decline in older adults.

**Methods:**

This is a systematic literature review registered on the International Prospective Register of Systematic Reviews (PROSPERO) and based on the criteria recommended by the Preferred Reporting Items for Systematic Reviews and Meta-Analyses (PRISMA). The Population, Intervention, Comparison, Outcome, and Study type (PICOS) strategy was used to define eligibility. Studies that met the criteria were included in the qualitative synthesis. We assessed the risk of bias through the Joanna Briggs Institute Critical Appraisal Checklists.

**Results:**

A total of 3,239 articles, found in eight databases, addressed the relationship between HA, CI, and cognition. We selected 30 experimental articles reporting measures of cognitive outcomes for older adults to include in the qualitative analysis. Of those, 23 studies reported a significant improvement in outcome and seven reported no significant change.

**Conclusions:**

This systematic review indicates that CI and HA can bring benefits to cognition in older adults.

**Systematic review registration:**

https://www.crd.york.ac.uk/prospero/display_record.php?ID=CRD42021273690

## Introduction

Over 1.5 billion people are estimated to experience some decline in hearing capacity during their lifetime, of which at least 430 million will need care (1). Hearing impairment is the third most common chronic disease that affects older adults and is present in about 30% of individuals aged 65 years or more and up to 90% of those are older than 85 years (2). When a person has dementia associated with hearing loss, the communication difficulties generated by both conditions are intensified. This can lead to consequences such as increased self-perception of advanced age, social isolation, reduced participation in daily life activities, and lower quality of life (3). Another important domain of mental health is reported to be affected by hearing loss is cognitive functioning itself (4), establishing a possible cause–effect nexus between the two conditions.

Dementia affects around 46.8 million people worldwide, resulting in a very high estimated financial cost (5). Current drug treatments targeting neuropathological processes such as Alzheimer's disease offer limited benefit. Studies indicate that presbycusis precedes the onset of clinical dementia by 5 to 10 years, being a possible non-invasive biomarker that may offer an alternative way to modify the management of these patients (5, 6).

The pathological mechanisms for hearing loss leading to cognitive decline remain unclear, although several hypotheses have been proposed. One of the main potential explanations is based on the “deprivation hypothesis,” according to which hearing loss affects the anatomic and functional integrity of the brain, as suggested by several magnetic resonance imaging (MRI) studies (7). Patients with untreated hearing loss often develop atrophy of the temporal lobe (auditory cortex), whereas patients with dementia have diffuse brain atrophy. In addition, cerebral gliosis has been demonstrated in both diseases, which may contribute to their symptoms (8).

Other mechanisms for this relationship include the “cognitive load hypothesis,” in which cognitive impairment could be the result of devoting more resources to effortful sensory perception, to the detriment of other cognitive processes; the “information degradation hypothesis,” that is when impoverished perceptual input causes cognitive decline; and the “common cause hypothesis,” which assumes that both hearing loss and cognitive impairment are caused by common underlying processes (e.g., generalized effects of the aging brain or age-related cerebrovascular disease). Although presented as alternatives, the different hypotheses proposed may not be mutually exclusive and are likely to act in conjunction (7).

Accordingly, if hearing loss does contribute to cognitive impairment, it could be expected that auditory rehabilitation, through HA or CI, should mitigate cognitive decline (2). However, there are no consistent answers to this question in the scientific literature thus far. Thus, this review aimed to assess whether HA or CI benefits cognition or mitigates cognitive decline in older adults with hearing loss.

## Methods

### Design and registration

This study is a systematic review, conceived according to the Cochrane Handbook for Systematic Reviews of Interventions (9) and registered in PROSPERO (International Prospective Register of Systematic Reviews) under the ID number CRD42021273690 (10). We conducted the research following the Preferred Reporting Items for Systematic Reviews and Meta-Analyses (PRISMA) methodology (11).

### Eligibility criteria

#### Inclusion criteria

We included studies following the PICOS strategy (anagram in which P denoted population; I, intervention (or exposure); C, comparison; O, outcome; and S, study type) (11). Our population of interest was “older adults with hearing loss,” the intervention “hearing rehabilitation through CI or HA,” and the comparison between “non-rehabilitated and rehabilitated patients;” the outcome was “improvement in cognition status,” and the study designs included randomized clinical trials, quasi-experimental studies, cohort studies, and systematic reviews with or without meta-analysis.

#### Exclusion criteria

(1) Age <50 years;

(2) Hearing rehabilitation through mechanisms other than CI or HA (e.g., stapedotomy, osteoanchored prosthesis, and ventilation tube);

(3) Studies in languages other than English, Portuguese, Spanish, or French.

### Search strategy

The search used Medical Subject Headings (MeSH) vocabulary in English and its alternative descriptors. After three pilot trials in the PubMed database to assess the feasibility of the proposal, the keywords and the search strategy were defined using Boolean operators in the following search string: “(Cochlear Implantation OR Cochlear Implants OR Cochlear Implant OR Hearing Aids OR Correction of Hearing Impairment OR Audiologic Rehabilitation OR Correction of Hearing Loss OR Rehabilitation of Hearing Impaired) AND (Cognitive Impairment OR dementia OR Cognitive Dysfunction OR Cognitive Decline OR Cognitive Dysfunctions OR Cognitive Impairment OR Neurocognitive Disorders OR Neurocognitive Dysfunction OR Cognitive Dysfunctions OR Mental Deterioration OR Alzheimer's disease)”.

The ultimate search swept eight electronic databases: PubMed (MEDLINE), SciELO, Scopus, Lilacs, Embase, Epistemonikos, ClinicalTrials.gov, and Cochrane Library, from September to October 2021. A subsequent update was performed in March 2022 to ensure up-to-date information.

### Data extraction

The initial search found 3,239 references, exported to EndNote Web (Clarivate Analytics) for reference management and deletion of duplicates. We excluded 772 duplicated articles, and the 2,467 unique references remaining were exported to the software Rayyan—Intelligent Systematic Review (https://www.rayyan.ai/) (12), allowing the selection process by two blinded reviewers (two different members of the research team, which gave their evaluations without knowledge of the other one's assessment). Initial evaluation of relevance was through the titles, then abstracts, and in the third stage, full-text analysis. In case of conflict, a third examiner decided whether or not to include the study.

We extracted data from the selected studies including authors, title, city/country of origin, year of publication, study design, number of participants, age, assessment method for hearing loss, cognitive domains evaluated, rehabilitation strategy, the time between assessments, and clinical outcomes.

### Risk of bias

Studies that met all of the inclusion criteria and were ultimately selected by our peer review were then evaluated by a third party through the Joanna Briggs Institute (JBI) Critical Appraisal Checklists (13) for systematic reviews, randomized clinical trials, and quasi-experimental studies (non-randomized), to assess the risk of bias. In our search, most of the studies that evaluated patients exposed to HA or CI did not classify themselves methodologically into either “cohort studies” or “quasi-experimental studies.” On the contrary, they mostly only mentioned that they were longitudinal studies that evaluated patients with and without auditory rehabilitation through HA or CI. Because our study searched for rehabilitation and both HA and CI are types of interventions, the follow-up after intervening in a non-randomized way would be considered a quasi-experimental study. Therefore, those studies were all evaluated using the JBI Critical Appraisal Checklist for quasi-experimental studies. The details of the items present in the checklists can be found in **Tables 5**, **6**, **7**.

### Ethical aspects

The methodological design in this study used secondary data analysis and was conducted by using a thorough process reinforcing research ethics in all of its stages and was approved by the Research Ethics Committee, in a national and unified database of research involving human beings CEP/CONEP, through the protocol number CAAE 36929420.1.0000.5082.

## Results

### Study selection

**Figure 1:**
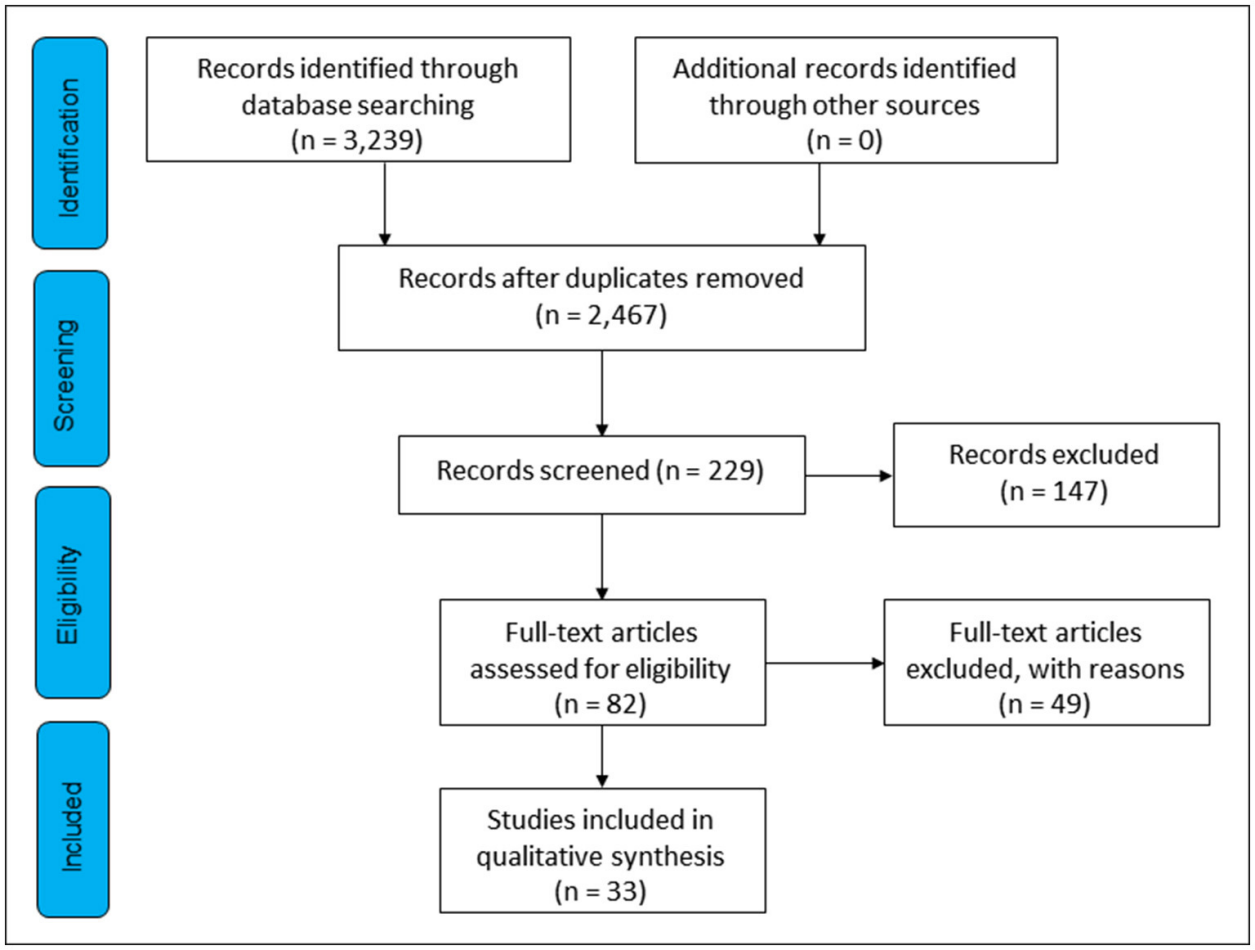
PRISMA flow diagram representing the selection process.

We selected 33 references, three of which corresponded to systematic review studies. No extra articles were found by searching the reference lists of included articles. The PRISMA flow diagram (Figure 1) details the article selection process.

### Characteristics and quality of the studies

The descriptive analysis of the studies was divided according to the type of study: “randomized clinical trials” (Table 1), “quasi-experimental and cohort” (Table 2), and “systematic reviews” (Table 3). The highlights of the cognitive outcome by intervention subgroup and study type are available in Table 4.

**Table 1:** Descriptive synthesis of the randomized clinical trials.

**Author, place and year**	**Title**	**Study design**	**Number and participants characteristics**	**Age**	**Auditory evaluation**	**Cognitive evaluation**	**Type of hearing intervention**	**Follow up**	**Outcome**
Karawani et al. (14) College Park, United States	Neural and behavioral changes after the use of hearing aids	Randomized Controlled Clinical Trial	55 HL with no signs of cognitive decline	60–84 years old	Pure Tone Audiometry	QuickSIN, ABR	HA	Before and 6 months after	Individuals that didn't use HA performed worse in temporal abilities and showed bigger physiological deficits in sound codification and neural synchrony after 6 months when compared to HA users
Karawani et al. (15) College Park, United States	Restoration of sensory input may improve cognitive and neural function	Randomized Controlled Clinical trial	50 HL with no signs of cognitive decline	60–84 years old	Pure Tone Audiometry	NIHTB-CFB	HA	Before and 6 months after	The use of HA for 6 months improved work memory and increased neural cortex processing
Nguyen et al. (16) Decines Charpieu, France	Efficacy of hearing aids on the cognitive status of patients with Alzheimer's disease and hearing loss: a multicenter controlled randomized trial	Randomized Double-blind Clinical Trial	51 HL and Alzheimer's Diagnosis	68–99 years old	Pure Tone Audiometry	DSM IV criteria for dementia, NINCDS-ADRDA e MMSE	HA	Before, 6 and 12 months after	No significant effect was found in cognition after 6 months in patients with Alzheimer's disease and HL
Nkyekyer et al. (17) Melbourne, Australia	The cognitive and psychosocial effects of auditory training and hearing aids in adults with hearing loss	Randomized Controlled Clinical Trial	40 HL with no signs of cognitive decline	50–90 years old	Pure Tone Audiometry	SUCCAB	HA	Before, 3 and 6 months after	HA reduced communication problems but there was no significant improvement in cognition

HL, Hearing loss; QuickSIN, Quick Speech in Noise; ABR, auditory brainstem response; NIHTB-CFB, NIH Toolbox Cognitive Function Battery; DSM IV, 4^th^ Diagnostic and Statistical Manual of Mental Disorders; NINCDS-ADRDA, National Institute of Neurological and Communicative Disorders and Stroke—Alzheimer's Disease and Related Disorders Association; MMSE, Mini-Mental State Examination; SUCCAB, Swinburne University Computerized Cognitive Assessment Battery; HA, Hearing aids.

**Table 2:** Descriptive synthesis of the quasi-experimental studies.

**Author, place and year**	**Title**	**Study design**	**Number and participants characteristics**	**Age**	**Auditory evaluation**	**Cognitive evaluation**	**Type of hearing intervention**	**Follow up**	**Outcome**
Acar et al. (18) Ankara, Turkey	Effects of hearing aids on cognitive functions and depressive signs in elderly people	Quasi-experimental	34 HL with no signs of cognitive decline	> 65 years old	Pure tone audiometry	MMSE	HA	Before, 6 months after	An increased cognitive function was observed after 6 months of HA use
Allen et al. (19) Manchester, United Kingdom	The effects of improving hearing in dementia.	Quasi-experimental	83 HL with cognitive decline	67–96 years old	Pure tone audiometry	MMSE	HA	Before, 1, 3 and 6 months after	Hearing improvement did not benefit cognitive function
Amieva et al. (2) Bordeaux, France	Self-reported hearing loss, hearing aids, and cognitive decline in elderly adults: a 25-year study	Quasi-experimental	3,777 HL with no signs of cognitive decline	> 65 years old	Self-assessment of hearing	MMSE	HA	Before, 25 years after	Patients with non-rehabilitated HL showed more cognitive deterioration than the control group. Individuals with HL using HA had no significant difference in cognitive decline compared to healthy controls
Castiglione et al. (20) Padua, Italy	Aging, cognitive decline and hearing loss: effects of auditory rehabilitation and training with hearing aids and cochlear implants on cognitive function and depression among older adults	Quasi-experimental	125 HL with no signs of cognitive decline	> 65 years old	Pure tone audiometry and vocal	MoCA, digit span e stroop test	HA or cochlear implant	Before, 1 and 12 months after	Positive effects were found with the use of HA or CI combined with auditory training on short-term memory. In the CI group, there was also a significant positive effect on cognitive performance after 1 year
Choi et al. (21) Seoul, Korea	Is cognitive function in adults with hearing impairment improved by the use of hearing aids?	Quasi-experimental	18 HL with no signs of cognitive decline	69.5 ± 8.3 years old	Pure tone audiometry and words in noise	Korean visual verbal learning test	HA	Before, 6 months after	Statistically significant differences in VVLT scores were found 6 months after hearing aid use, but not in the control group
Claes et al. (22) Ghent, Belgium	Cognitive performance of severely hearing-impaired older adults before and after cochlear implantation: preliminary results of a prospective, longitudinal cohort study using the RBANS-H	Quasi-experimental	20 HL with no signs of cognitive decline	54–84 years old	Pure tone audiometry	RBANS-H	Cochlear implant	Before, 6 and 12 months after	Significant improvement in general cognition after 12 months of CI use, which can be attributed to significant improvement in immediate and delayed memory and, to a lesser extent, changes in working memory, processing speed, and sustained attention
Cosetti et al. (23) Shreveport, Baltimore e New York, United States	Neurocognitive testing and cochlear implantation: insights into performance in older adults	Quasi-experimental	7 HL with no signs of cognitive decline	67–81 years old	Pure tone audiometry	TOPF + WASI + TMT + Controlled Oral Word Association Tests + BNT + RBANS	Cochlear implant	Before, 2 to 4 years after	Improvement in cognitive function over time in older adults with CI
Cuoco et al. (24) Salerno, Italy	Neuropsychological profile of hearing-impaired patients and the effect of hearing aid on cognitive functions: an exploratory study	Quasi-experimental	56 HL with no signs of cognitive decline	> 55 years old	Pure tone audiometry and vocal	MoCA + DCT + TMT-A, 15-RAWLT, ROCF memory, ROCF copy, CDT e COM	HA	Before, 6 months after	No difference was found between patients that used or not HA in the cognitive tests
Dawes et al. (25) Manchester, United Kingdom e Madison, United States	Hearing-aid use and long-term health outcomes: hearing handicap, mental health, social engagement, cognitive function, physical health, and mortality	Quasi-experimental	4541 HL with no signs of cognitive decline	48–92 years old	Pure tone audiometry	MMSE, TMT, DSST, AVLT e VFT	HA	Before, 11 years after	There was no difference in cognitive performance or the incidence of cognitive impairment between hearing aid users and non-users
Deal et al. (26) Baltimore, EUA	Hearing impairment and cognitive decline: a pilot study conducted within the atherosclerosis risk in communities neurocognitive study	Quasi-experimental	253 Normal hearing or HL with no signs of cognitive decline	≥ 45 years old	Pure tone audiometry	DWRT, Word Fluency Test e DSST	HA	Before, 4 and 23 years after	Comparing participants with moderate/severe HL with participants without HL, the 20-year rates of decline in memory and global function differed and were higher in participants who did not wear hearing aids
Glick and Sharma (27) Boulder, United States	Cortical neuroplasticity and cognitive function in early-stage, mild-moderate hearing loss: evidence of neurocognitive benefit from hearing aid use	Quasi-experimental	41 HL with no signs of cognitive decline	64 ± 4.68 years old	Pure tone audiometry and vocal	MoCA, BDS-2, SDMT, RST e WARRM	HA	Before, 6 months after	HA reversed modal recruitment of the auditory cortex for visual processing after 6 months, coinciding with gains in speech perception skills and improvements in global cognitive function, executive functions, processing speed and visual working memory performance
Jayakody et al. (28) Carlton, Australia	Impact of cochlear implantation on cognitive functions of older adults: pilot test results	Quasi-experimental	39 HL with no signs of cognitive decline	69.04 ± 12.35 years old	Pure tone audiometry	CANTAB + AST + PAL + VRM + RTI + SWM	Cochlear implant	Before, 6 and 12 months after	Significant improvement in spatial working memory, strategies and simple RTI tasks after 6 months. After 12 months there was a positive impact on cognitive flexibility, PAL, SWM, strategies and simple RTI Tasks
Luz et al. (29) São Paulo, Brazil	Restrictions in participation and mental state in new hearing aids users	Quasi-experimental	50 HL with no signs of cognitive decline	>60 years old	Pure tone audiometry	MMSE	HA	Before and 12 to 16 weeks after	Improved cognitive processes of orientation in time and space, repetition/recording, attention, calculation and language
Magalhães et al. (30) São Paulo, Brazil	Evaluation of participation restriction and cognitive processes in the elderly before and after the audiologic rehabilitation	Quasi-experimental	50 HL with no signs of cognitive decline	>60 years old	Pure tone audiometry e SRT	MMSE	HA	Before and about 1 year after	Better results in cognitive processes after speech therapy, regardless of gender and age variables
Maharani et al. (31) Manchester, United Kingdom	Longitudinal relationship between hearing aid use and cognitive function in older americans	Quasi-experimental	2040 HL with no signs of cognitive decline	>50 years old	Pure tone audiometry	Assessment of episodic memory (immediate and delayed recall of words).	HA	Before and 18 years after	Slower decline in memory performance in participants with HL after hearing aid
Mertens et al. (32) Antwerp, Belgium; Madrid, Spain; Warsaw, Poland; Bradford, United Kingdom; Perth, Australia	Cognitive improvement after cochlear implantation in older adults with severe or profound hearing impairment: a prospective, longitudinal, controlled, multicenter study	Quasi-experimental controlled and multicentric	48 HL with no signs of cognitive decline	> 55 years old	Pure tone audiometry	RBANS-H	Cochlear implant	Before, 14 months after	Improvements in general cognitive functioning and “Attention” compared to control
Mosnier et al. (33) Paris, France	Improvement of cognitive function after cochlear implantation in elderly patients	Quasi-experimental	94 HL with no signs of cognitive decline	65–85 years old	Pure tone audiometry	MMSE, 5-word test, clock-drawing test, verbal fluency test, d2 test of attention e Trail Making test parts A and B	Cochlear implant	Before, 6 and 12 months after	Better mean scores were found in all cognitive domains after 6 and 12 months of CI intervention. 81% of the CI group showed improved global cognitive function
Mosnier et al. (34) Paris, France	Long-term cognitive prognosis of profoundly deaf older adults after hearing rehabilitation using cochlear implants.	Quasi-experimental	70 HL with no signs of cognitive decline	> 65 years old	Pure tone audiometry	MMSE, 5-word test, clock-drawing test, verbal fluency test, d2 test of attention e Trail Making test parts A e B	Cochlear implant	Before, 1, 5 years or more after	A lower rate of progression to dementia was observed in the post CI follow-up and also improvements in cognitive function in some individuals
Sarant et al. (35) Melbourne, Australia	The effect of hearing aid use on cognition in older adults: can we delay decline or even improve cognitive function?	Quasi-experimental	99 HL with no signs of cognitive decline	61–84 years old	Pure tone audiometry	MMSE, Cogstate Brief Battery e GMLT of executive function	HÁ	Before and 18 months after	The rehabilitated group's mean scores on cognitive tests showed no decline and executive function improved significantly. Clinically significant improvement or stability in executive function was found for 97.3% of participants. Women also improved working memory, visual attention and visual learning
Sarant et al. (36) Melbourne, Australia	The effect of cochlear implants on cognitive function in older adults: initial baseline and 18-month follow up results for a prospective international longitudinal study	Quasi-experimental	59 HL with no signs of cognitive decline	61–89 years old	Pure tone audiometry	MMSE, cogstate brief battery e GMLT of executive function	Cochlear implant	Before and 18 months after	Significant improvements in executive function were observed for men without higher education after CI, while cognitive function did not decline for the other participants
Sonnet et al. (37) Nancy, France	Cognitive abilities and quality of life after cochlear implantation in the elderly	Quasi-experimental	16 HL with no signs of cognitive decline	65 - 80 years old	Pure tone audiometry	MMSE	Cochlear implant	Before, 6 and 12 months after	Cognitive functions were not influenced by CI, but there was a benefit in executive functions
Tai et al. (38) Taiwan	Effects of hearing impairment and hearing aid use on the incidence of cognitive impairment among community-dwelling older adults: evidence from the Taiwan Longitudinal Study on Aging (TLSA)	Quasi-experimental	709 HL with no signs of cognitive decline	> 60 years old	Self-report of hearing impairment or previous use of HA	SPMSQ	HA	Before, 4, 8 and 12 years after	Patients with HL who used HA had a lower incidence of cognitive impairment (66.3% vs. 75.6%) compared to those who did not use HA during the 12-year follow-up. However, the adjusted odds ratio did not show significant decreases in hearing aid users when compared to non-users
Tesch-Römer et al. (39) Greifswald, Germany	Psychological effects of hearing aid use in older adults	Quasi-experimental	140 HL with no signs of cognitive decline	51 - 87 years old	Pure tone audiometry	Berlin aging study test modified	HA	Before and 6 months after	There was no effect of hearing aid use on cognitive functioning
Van Hooren et al. (40) Maastricht, Holand	Does cognitive function in older adults with hearing impairment improve by hearing aid use?	Quasi-experimental	102 HL with no signs of cognitive decline	> 60 years old	Pure tone audiometry	SCWT, CST, LDST, VVLT e verbal fluency test	HA	Before and 12 months after	Although participants with HA had improved hearing thresholds, they did not demonstrate better performance on cognitive tests compared to controls
Vasil et al. (41).	How Does Cochlear Implantation Lead to Improvements on a Cognitive Screening Measure?	Quasi-experimental	77 HL with no signs of cognitive decline	55–85 years old	Pure tone audiometry	MoCA	Cochlear implant	Before and 6 months after	Better performances were demonstrated in the MoCA test 6 months after CI
Völter et al. (42) Bochum, Germany	Can cochlear implantation improve neurocognition in the aging population?	Quasi-experimental	60 HL with no signs of cognitive decline	50 - 84 years old	Pure tone audiometry	Short–term and long-term memory, processing speed, attention, working memory and inhibition.	Cochlear implant	Before, 6 and 12 months after	Neurocognitive skills increased significantly after 6 months. At 12 months, most cognitive domains remained stable, except for working memory assessed by the span task, which improved significantly between 6 and 12 months

HL, hearing loss; MMSE, Mini-Mental State Examination; MoCA, Montreal Cognitive Assessment; RBANS, Repeatable Battery for the Assessment of Neuropsychological Functioning; TOPF, test of premorbid functioning; WASI, Wechsler Abbreviated Scale of Intelligence; TMT, trail making test; TMT-A, trail making test-part A; BNT, Boston Naming Test; DCT, digit cancellation test; 15-RAWLT, Rey's auditory 15-word learning test to assess verbal immediate recall and verbal delayed recall; ROCF memory, Rey–Osterrieth Complex Figure Test memory; CDT, clock drawing test; CPM,Raven's Colored Progressive Matrices; DSST, digit symbol substitution test; AVLT, auditory verbal learning test; VFT, verbal fluency test; DWRT, delayed word recall test; BDS-2, Behavioral Dyscontrol Scale II (executive function); SDMT, Symbol Digits Modalities Test (processing speed); RST, reading span test (visual working memory); WARRM, Word Auditory Recognition and Recall Measure (auditory working memory); CANTAB, Cambridge Neuropsychological Test Automated Battery; AST, attention switching task; PAL, paired-associate learning; VRM, verbal recognition memory; RTI, reaction time; SWM, spatial working memory; GMLT, Groton Maze Learning Test; SPMSQ, Short Portable Mental Status Questionnaire; SCWT,Stroop Color-Word Test; CST, Concept Shifting Task; LDST, Letter-Digit Substitution Test; VVLT, visual verbal learning test; HA, hearing aid; SRI, Speech Recognition Index.

**Table 3:** Descriptive synthesis of the systematic review studies.

**Author, place and year**	**Title**	**Study design**	**Number and participants characteristics**	**Age**	**Participant profile**	**Cognitive evaluation**	**Type of hearing intervention**	**Outcome**
Claes et al. (43) Ghent, Belgium	Cognitive outcomes after cochlear implantation in older adults: a systematic review	Systematic review	166 (6 studies)	> 50 years old	Patients with HL	Varied	Cochlear implant	Available studies do not provide conclusive evidence of better cognitive outcomes after cochlear implant in the older adults
Miller et al. (44) Tucson, United States.	The impact of cochlear implantation on cognition in older adults: a systematic review of clinical evidence	Systematic review	89 (3 studies)	≥ 65 years old	Patients with HL	Varied	Cochlear implant	Although many publications have shown that CI improves speech perception, social functioning and overall quality of life, we have not found studies in the literature that have prospectively evaluated changes in cognitive function after CI in the older adults
Utoomprurkporn et al. (45) London, United Kingdom.	Hearing-impaired population performance and the effect of hearing interventions on Montreal Cognitive Assessment (MoCA): systematic review and meta-analysis	Systematic review and meta-analysis	950 (12 studies)	≥ 60 years old	Patients with HL	MoCA	Cochlear implant or HA	There was no significant difference in MoCA scores between pre and post intervention (3 studies, *N* = 75). However, sensitivity analysis in cochlear implant studies (2 studies, *N* = 33) showed an improvement in the MoCA score of 1.73

HL, Hearing loss; MoCA, Montreal Cognitive Assessment; HA, Hearing aid.

**Table 4:** Cognitive improvement.

**Type of Study**	**Cognitive improvement**	**Number of studies**	**Sample size**	**% of the sample**
**HA**						
Randomized Clinical trials	Yes	2	105	53,57%
	No	2	91	46,43%
Quasi-experimental studies	Yes	11	7,196	59,38%
	No	5	4,922	40,62%
**CI**						
Randomized Clinical trials	Yes	0	0	0
	No	0	0	0
Quasi-experimental studies	Yes	10	599	97,39%
	No	1	16	2,61%

**Figure 2:**
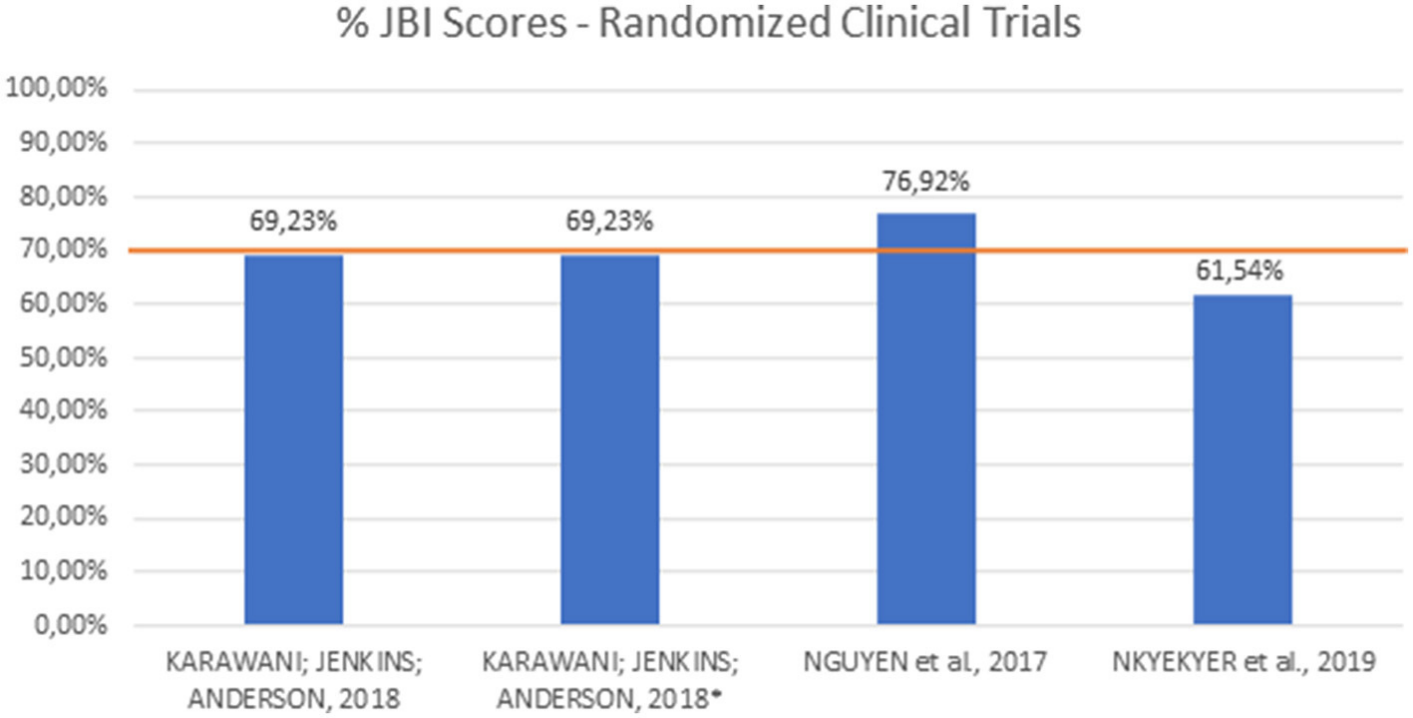
JBI scores for randomized clinical trials.

**Figure 3:**
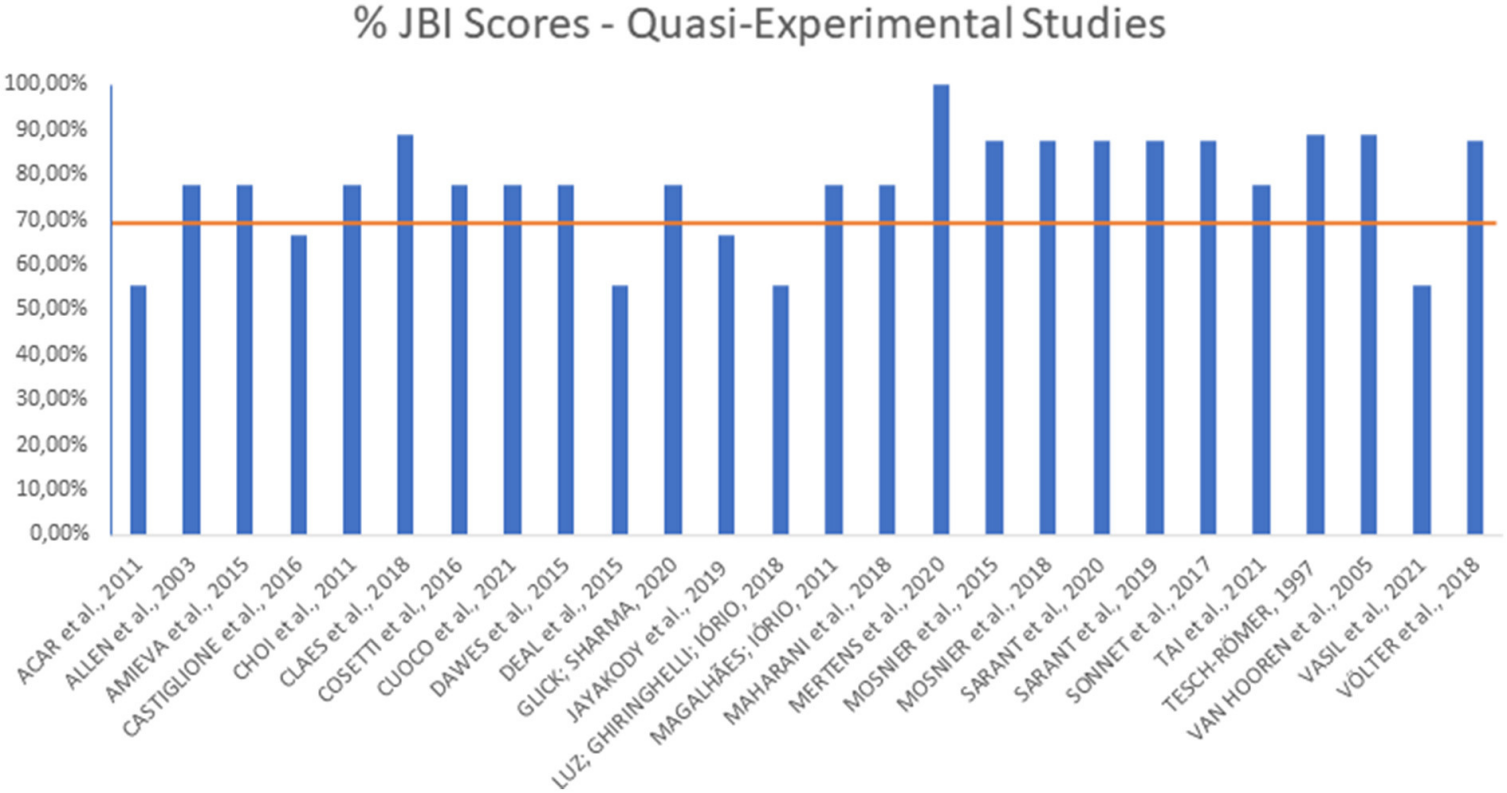
JBI scores for quasi-experimental studies.

A summary of the articles' quality regarding the JBI risk of bias assessment is given in Figures 2, 3. The majority of the included experimental studies obtained a percentage of adequacy to the criteria close to or above 70% and therefore considered, respectively, of high and moderate methodological quality. The mean score was 69.23% for randomized clinical trials and 77.88% for quasi-experimental studies. The complete measurement questions and answers in each kind of study are given in Tables 5, 6, 7.

**Table 5:** JBI Checklist for randomized clinical trials.

	**Karawani et al. (14)**	Karawani et al. (15)^*^	**Nguyen** **et al. (16)**	**Nkyekyer et al. (17)**
1. Was true randomization used for assignment of participants to treatment groups?	Yes	Yes	Yes	Yes
2. Was allocation to treatment groups concealed?	No	No	No	No
3. Were treatment groups similar at the baseline?	Yes	Yes	Yes	Yes
4. Were participants blind to treatment assignment?	No	No	Yes	No
5. Were those delivering treatment blind to treatment assignment?	No	No	No	No
6. Were outcomes assessors blind to treatment assignment?	No	No	No	No
7. Were treatment groups treated identically other than the intervention of interest?	Yes	Yes	Yes	Yes
8. Was follow up complete and if not, were differences between groups in terms of their follow up adequately described and analyzed?	Yes	Yes	Yes	No
9. Were participants analyzed in the groups to which they were randomized?	Yes	Yes	Yes	Yes
10. Were outcomes measured in the same way for treatment groups?	Yes	Yes	Yes	Yes
11. Were outcomes measured in a reliable way?	Yes	Yes	Yes	Yes
12. Was appropriate statistical analysis used?	Yes	Yes	Yes	Yes
13. Was the trial design appropriate, and any deviations from the standard RCT design (individual randomization, parallel groups) accounted for in the conduct and analysis of the trial?	Yes	Yes	Yes	Yes

* Title, Neural and behavioral changes after the use of hearing aids.

Table 6JBI Checklist for quasi-experimental studies.

**Table:** 

**Part 1**															
	**Acar et al. (18)**	**Allen et al. (19)**	**Amieva et al. (2)**	**Castiglione et al. (20)**	**Choi et al. (21)**	**Claes et al. (22)**	**Cosetti, et al. (23)**	**Cuoco et al. (24)**	**(25)**	**Deal, et al. (26)**	**Glick, Sharma, et al. (27)**	**Jayakody, et al. (28)**	**Luz; Ghiringhelli; Iório, et al. (29)**	**Magalhães; Iório, et al. (30)**	**Maharani, et al. (31)**
1. Is it clear in the study what is the ‘cause' and what is the ‘effect' (i.e., there is no confusion about which variable comes first)?	Unclear	Yes	Yes	Yes	Yes	Yes	Yes	Yes	Yes	Yes	Yes	Yes	Yes	Yes	Yes
2. Were the participants included in any comparisons similar?	Yes	Yes	Yes	No	No	No	Yes	Yes	Yes	Unclear	Yes	Yes	Yes	No	Yes
3. Were the participants included in any comparisons receiving similar treatment/care, other than the exposure or intervention of interest?	No	No	Unclear	No	Yes	Yes	No	No	No	Unclear	No	No	No	Yes	Unclear
4. Was there a control group?	No	No	Yes	Yes	Yes	Yes	No	Yes	No	Yes	No	No	No	No	No
5. Were there multiple measurements of the outcome both pre and post the intervention/exposure?	No	Yes	Yes	No	No	Yes	Yes	No	Yes	Yes	Yes	No	No	Yes	Yes
6. Was follow up complete and if not, were differences between groups in terms of their follow up adequately described and analyzed?	Yes	Yes	Yes	Yes	Yes	Yes	Yes	Yes	Yes	Yes	Yes	Yes	Yes	Yes	Yes
7. Were the outcomes of participants included in any comparisons measured in the same way?	Yes	Yes	Yes	Yes	Yes	Yes	Yes	Yes	Yes	No	Yes	Yes	Yes	Yes	Yes
8. Were outcomes measured in a reliable way?	Yes	Yes	Unclear	Yes	Yes	Yes	Yes	Yes	Yes	Unclear	Yes	Yes	Yes	Yes	Yes
9. Was appropriate statistical analysis used?	Yes	Yes	Yes	Yes	Yes	Yes	Yes	Yes	Yes	Yes	Yes	Yes	Unclear	Yes	Yes

**Table:** 

**Part 2**											
	**Mertens et al. (32)**	**Mosni, er et al. (33)**	**Mosnier, et al. (34)**	**Sarant, et al. (35)**	**Sarant, et al. (36)**	**Sonnet, et al. (37)**	**Tai**, **et al. (38)**	**Tesch-römer, et al. (39)**	**Van hooren**, **et al. (40)**	**Vasil**, **et al. (41)**	**Völter, et al. (41)**				
1. Is it clear in the study what is the ‘cause' and what is the ‘effect' (i.e. there is no confusion about which variable comes first)?	Yes	Yes	Yes	Yes	Yes	Yes	Yes	Yes	Yes	Yes	Yes				
2. Were the participants included in any comparisons similar?	Yes	N/A	N/A	N/A	N/A	N/A	Yes	Yes	Unclear	Unclear	N/A				
3. Were the participants included in any comparisons receiving similar treatment/care, other than the exposure or intervention of interest?	Yes	Yes	Yes	Yes	Yes	Yes	Yes	Yes	Yes	Unclear	Yes				
4. Was there a control group?	Yes	No	No	No	No	No	Yes	Yes	Yes	No	No				
5. Were there multiple measurements of the outcome both pre and post the intervention/exposure?	Yes	Yes	Yes	Yes	Yes	Yes	Yes	Yes	Yes	Yes	Yes				
6. Was follow up complete and if not, were differences between groups in terms of their follow up adequately described and analyzed?	Yes	Yes	Yes	Yes	Yes	Yes	Yes	Yes	Yes	Yes	Yes				
7. Were the outcomes of participants included in any comparisons measured in the same way?	Yes	Yes	Yes	Yes	Yes	Yes	Unclear	Yes	Yes	No	Yes				
8. Were outcomes measured in a reliable way?	Yes	Yes	Yes	Yes	Yes	Yes	Unclear	Yes	Yes	Yes	Yes				
9. Was appropriate statistical analysis used?	Yes	Yes	Yes	Yes	Yes	Yes	Yes	Unclear	Yes	Yes	Yes				

**Table 7:** JBI Checklist for systematic literature reviews.

	**Claes et al. (22)**	**Miller et al. (44)**	**Utoomprurkporn et al. (45)**
1. Is the review question clearly and explicitly stated?	Yes	Yes	Yes
2. Were the inclusion criteria appropriate for the review question?	Yes	No	Yes
3. Was the search strategy appropriate?	Yes	Yes	Yes
4. Were the sources and resources used to search for studies adequate?	No	Yes	Yes
5. Were the criteria for appraising studies appropriate?	Yes	No	Yes
6. Was critical appraisal conducted by two or more reviewers independently?	Yes	Yes	Yes
7. Were there methods to minimize errors in data extraction?	Unclear	Unclear	Yes
8. Were the methods used to combine studies appropriate?	Yes	Yes	Unclear
9. Was the likelihood of publication bias assessed?	Yes	Unclear	Yes
10. Were recommendations for policy and/or practice supported by the reported data?	No	Unclear	Unclear
11. Were the specific directives for new research appropriate?	Yes	No	Yes

## Discussion

The global increase in life expectancy is one of the greatest health achievements in the last 150 years. However, age-related chronic conditions have also increased substantially, negatively affecting the independence and quality of life of older adults, contributing to a pessimistic picture of aging, in which presbycusis plays a leading role (4). Associated with the aging of the population, there is also a progressive increase in dementia and neurocognitive disorders (46). This research sought to systematically analyze the literature on the cognitive benefits of treating hearing loss, to determine the best management practices in this vulnerable and growing population.

### Patient profile

Only two studies (16, 19) searched a population with hearing loss associated with signs of cognitive decline and/or diagnosis of Alzheimer's disease; two other studies (33, 34) included hearing loss patients with or without cognitive alterations. The remaining studies selected patients with hearing loss without signs of cognitive disease.

As for the age of the patients, most studies evaluated individuals over 60 years. In total, three studies (24, 32, 41) included participants aged 55 and older, and another three studies (17, 31, 39) included 50 or older patients. Because age acts as a risk factor for both presbycusis and dementia, a clear definition of age groups was fundamental to this review.

### Main outcome

#### Type of intervention: Cochlear implant

In 10 of the articles, the intervention was based on cochlear implants, including a total of 615 CI recipients. Of these, only one study (37) found no difference in the cognitive functions after CI, although there was a benefit on executive functions (responsible for coordination and integration of the neurofunctional triad of learning: working memory, inhibitory control, and cognitive flexibility). This study evaluated patients prospectively, through the Mini-Mental State Examination (MMSE), before surgery, 6 months after, and 12 months after the CI, with a sample of 16 patients. All the other studies reported improvements in cognitive domains after CI.

Castiglione et al. (20) found that among patients undergoing CI, there was a statistically significant positive effect on cognitive performance after 1 year. The analysis of the Montreal Cognitive Assessment (MoCA) subtasks showed that the greatest increment after treatment and auditory training was in long-term memory, followed by tasks that assess visual–spatial and logical executive skills. We must point out that MoCA needs to be adapted with a visual on-screen presentation for patients with hearing loss, to avoid bias related to their hearing impairment. In this study, the authors reported that the test materials were adapted for individuals according to their hearing loss and hearing treatment program.

Claes et al. (22) conducted a quasi-experimental study in which they evaluated older patients with severe post-lingual hearing loss preoperatively, and 6 and 12 months after rehabilitation with CI, using the Repeatable Battery for the Assessment of Neuropsychological Functioning for Hearing impaired individuals (RBANS-H). This battery of cognitive tests is specific for the hearing impaired, a strength of this study. The authors found a significant improvement in general cognition after 12 months, which could be attributed to the increase in immediate and delayed memory and, to a lesser extent, also to changes in working memory, processing speed, and sustained attention. Even though the study reported preliminary results, with a sample of only 20 patients, it showed promising outcomes.

Mertens et al. (32) conducted a prospective, quasi-experimental controlled, and multicenter study with a similar approach, using the RBANS-H, with a larger sample, of 48 patients. The patients were evaluated before and 14 months after CI, with improvements in general cognitive functioning and in the “attention” subdomain, compared to the control group.

Similar results were found in two other studies (28, 29) that used two different complex batteries of neurocognitive assessments. Cosetti et al. (23) had a slightly longer follow-up than all other studies in this group, ranging from 2 to 4 years (mean 3.7 years). Jayakody et al. (28) described that the cognitive tests used were adapted for patients with hearing loss.

Overall, two publications from the same research group (33, 34) had the same design but changed in the sample size (94 and 70 participants, respectively) and in the follow-up time (12 months and 5 years, respectively). A multi-domain cognitive assessment showed that 6 months after CI, there were better mean scores in all cognitive domains, and after 12 months, 81% of the intervention group showed improvement in global cognitive function. After 5 years, a possible beneficial effect on cognition was also demonstrated. Of the patients with prior mild cognitive decline, 6% developed dementia, 61% remained stable, and 32% returned to normal cognition. Among patients with normal cognition, 32% developed mild cognitive decline and none developed dementia.

The study conducted by Sarant et al. (36) had a follow-up time of 18 months and a sample size of 59 patients. The authors observed relative stability and statistically significant clinical improvement in cognition after the intervention. Völter et al. (42) found that 6 months after CI, neurocognitive skills increased significantly and between 6 and 12 months, and most cognitive domains remained stable, except for working memory (assessed by the span task), which improved.

#### Type of intervention: AASI

Overall, 20 articles had HA as an intervention, including a total of 12,149 fitted patients. Of these, seven studies found no benefit for cognition in the use of hearing aids. (16, 17, 19, 24, 25, 39, 40). The remaining 13 studies (2, 14, 15, 18, 20, 21, 26, 27, 29–31, 35, 38), which accounted for approximately 60% of the patient sample, obtained positive responses after HA intervention.

For the definition of HL in the target population, the studies considered audiometric parameters, necessarily including pure-tone audiometry (PTA) thresholds, with the exception of two studies. Amieva et al. (2) and Tai et al. (38) defined hearing loss through patients' self-report, without presenting objective criteria, which could have generated an information bias of the instrument kind (in which the measurement instrument provides inadequate results).

One study (20) assessed simultaneously HA and CI. The data reported indicate a positive effect of hearing aids or CI combined with auditory training on short-term memory in both groups. Among patients who underwent CI surgery, auditory rehabilitation also resulted in a positive effect on cognitive performance after 1 year.

The only two groups that studied the outcome in populations of patients with signs of cognitive illness or a diagnosis of dementia (16, 19) found no significant effect of hearing aid use on cognition after 6 months of treatment, suggesting that hearing improvement did not benefit cognitive function in this specific profile of patients.

### Follow-up time

A total of four studies maintained longer patient follow-ups (2, 25, 26, 31), all in the HA group; three of them (2, 26, 31) also had the largest sample sizes, adding up to 10,358 patients together. Amieva et al. (2) had the longest follow-up of 25 years; followed by Deal et al. (26), with 23 years; Maharani et al. (31), with 18 years; and Dawes et al. (25), with 11 years.

Some studies, notably those in which cognitive tests were repeated three times in 1 year (22, 28, 33, 34, 37, 42), could have been susceptible to “practice effect” bias, in which the performance improvement may be also due to a learning process of the patients.

Furthermore, in general, studies with CI had shorter follow-ups, which could be explained by the fact that this technology was not so available in the past and has achieved a greater reach in the last few years. However, as cognitive decline and dementia are both slowly progressive phenomena,^4^ we believe that there is still a lack of studies with longer follow-ups (of several years or decades), especially in the CI group.

### Additional findings

Sensory deprivation in older adults has been associated with decreased daily life activity and social participation, quality of life losses, and depressive symptoms. There is also a negative impact on individuals' autonomy due to a greater dependence on the caregivers (47). In addition to cognition impacts, some of the studies in this systematic review assessed additional outcomes. There was a general trend of positive correlations, indicating improvements to these outcomes after hearing rehabilitation. However, one study (19) did not observe improvement in daily life activities, psychiatric symptoms, or caregiver burden after the intervention.

Acar et al. (18), Castiglione et al. (20), Jayakody et al. (28), Mosnier et al. (34) and Nkyekyer et al. (17) found a positive correlation between auditory intervention and the reduction of depression levels, using the Geriatric Depression Scale (GDS). These results agreed with other studies in the scientific literature (48–50).

It is argued that there is also an indirect link mediated by social isolation, reduced physical activity, and depressive symptoms, which would also act as risk factors for dementia (4). These additional outcomes become even more relevant in this discussion when the “cognitive load hypothesis” is introduced as an alternative explanation for the cognitive decline in older adults with HL. This hypothesis is based on the principle that cognitive impairment could also result from the dedication of large brain resources to sensory perception to the detriment of other cognitive processes. Although presented as alternatives, the different hypotheses may not be mutually exclusive and probably work together in etiopathogenesis (4).

The assessment of the quality of life (QoL) has gained ground in the latest research. A total of six CI studies also evaluated the QoL as an outcome, through questionnaires such as “World Health Organization–Quality of Life” (WHOQOL). Only one research found stable QoL scores from 1 to 7 years after CI (34). All the others encountered a positive and significant QoL effect (22, 35–37, 42).

In addition, four studies found improvements in the self-perception of HL and participation restriction after auditory rehabilitation (29, 30, 32, 39). Among the questionnaires for this assessment, the Hearing Handicap Inventory for the Elderly (HHIE) and its shortest version, the Hearing Handicap Inventory for the Elderly–Screening Version (HHIE-S), stand out. These forms are composed of two scales: social, assessing the impact of hearing loss on the activities performed by the individual, and emotional, measuring the emotional response to hearing loss (51).

### Study designs

Of the 30 studies included in the descriptive synthesis of this systematic review, only four were randomized clinical trials (14–17), as shown in Table 1. Many of the authors (2, 20–22, 24, 26, 32, 38, 39), despite conducting prospective and controlled studies, opted not to randomize them, pledging the existence of ethical barriers preventing their course. Therefore, their studies were categorized as quasi-experimental. According to these authors, although a randomized clinical trial is always the preferred option in terms of study design, it would be unethical to deny treatment to a population with significant hearing loss, especially when the intervention in the discussion was HA, as they are considered to be innocuous. Thus, the control groups of these studies were formed by patients who did not want the intervention.

### Comparison with other systematic reviews

A total of three systematic reviews (43–45) presented themes and methodology comparable to this study and were therefore included and critically assessed. Claes et al. (43) conducted a study that evaluated patients whose rehabilitation was performed with CI. As for the databases used, there was a limited search, which included only two databases: MEDLINE (PubMed) and Cochrane Library. They included six studies, with a sample size of 166 patients. Although five of the six studies reported improvement in cognition after CI, in a variety of cognitive domains, the results were exposed to various risks of bias and were therefore considered to be inconclusive by the authors themselves.

Miller et al. (44) selected only three studies that met the inclusion criteria for the review, according to the authors. However, the age range of the three studies also included younger patients, 14, 19, and 23 years, respectively. In addition, one of the three studies described neither the measurements used for cognitive outcome results nor the number of participants in the sample, according to the table described in the authors' results, which could impair the reliability of the study.

Utoomprurkporn et al. (45) conducted a systematic review with meta-analysis including studies that reported MoCA score results for individuals with HL before and after CI intervention. A total of three studies met the criteria, with a sample of 75 patients. No significant difference in MoCA scores between pre- and post-rehabilitation was found. However, sensitivity analysis of two studies (with a sample of 33 individuals) showed an improvement in the MoCA score of 1.73. Despite achieving greater heterogeneity by selecting a single cognitive evaluation test (MoCA), this study reduced the sample size and, consequently, its length. Currently, a large number of different cognitive assessment tests were used, and their inclusion may help the extrapolation of the results data.

Our systematic review included 30 articles, whose methodological quality was considered high and moderate, with a total sample size of 12,804 patients, enabling the conclusion that auditory rehabilitation with CI or HA has a positive impact on cognition, leading to improvements in cognitive parameters.

## Conclusion

The results of this systematic review indicate that in older adults with hearing loss, the use of CI or HA can bring benefits to cognition and are therefore a promising strategy in the rehabilitation field, not only for hearing abilities but also for cognitive status improvement.

## Data availability statement

The original contributions presented in the study are included in the article/supplementary materials, further inquiries can be directed to the corresponding author.

## Author contributions

NC planned the methodological design and performed the search in all databases, was one of the two reviewers blinded through inclusion steps, and wrote the Discussion section, and thus has the first authorship. PL oversaw the blinding process and the methodological progress and helped with the theoretical basis of the discussion, and has the second authorship. IM was the second blinded reviewer and helped construct the result tables, and thus has the third authorship, MB was the third part judge in the decisions on whether to include articles when a consensus could not be reached by the two blind reviewers and thus has the fourth authorship. HR and CC contributed equally to the discussions and suggested improvements throughout the manuscript, and FB was the senior advisor who supervised and oriented the entire production process of this systematic review and thus has senior and corresponding authorship. All authors contributed to the article and approved the submitted version.

## Conflict of interest

The authors declare that the research was conducted in the absence of any commercial or financial relationships that could be construed as a potential conflict of interest.

## Publisher's note

All claims expressed in this article are solely those of the authors and do not necessarily represent those of their affiliated organizations, or those of the publisher, the editors and the reviewers. Any product that may be evaluated in this article, or claim that may be made by its manufacturer, is not guaranteed or endorsed by the publisher.
